# Dual Role of MicroRNAs in NAFLD

**DOI:** 10.3390/ijms14048437

**Published:** 2013-04-17

**Authors:** Sara Ceccarelli, Nadia Panera, Daniela Gnani, Valerio Nobili

**Affiliations:** Hepato-Metabolic Disease Unit and Liver Research Unit, Bambino Gesù Children’s Hospital, IRCCS, Piazza S. Onofrio, 4, 00165 Rome, Italy; E-Mails: nadia.panera@opbg.net (N.P.); daniela.gnani@yahoo.it (D.G.); nobili66@yahoo.it (V.N.)

**Keywords:** microRNAs, NAFLD, NASH, hepatic fibrosis

## Abstract

MicroRNAs are important post-transcriptional regulators in different pathophysiological processes. They typically affect the mRNA stability or translation finally leading to the repression of target gene expression. Notably, it is thought that microRNAs are crucial for regulating gene expression during metabolic-related disorders, such as nonalcoholic fatty liver disease (NAFLD). Several studies identify specific microRNA expression profiles associated to different histological features of NAFLD, both in animal models and in patients. Therefore, specific assortments of certain microRNAs could have enormous diagnostic potentiality. In addition, microRNAs have also emerged as possible therapeutic targets for the treatment of NAFLD-related liver damage. In this review, we discuss the experimental evidence about microRNAs both as potential non-invasive early diagnostic markers and as novel therapeutic targets in NAFLD and its more severe liver complications.

## 1. Introduction

MicroRNAs (miRNAs or miRs) are endogenous, small non-coding RNAs which possess a central role in the regulation of both mRNA and protein expression of the target genes. They were firstly described in *Caenorhabditis elegans* as regulators of developmental timing [1,2]. The miRNAs act at post-transcriptional level as ~20–30 nucleotides targeting the 3′-untranslated regions (3′-UTRs), which typically contain defined stability elements (including miRNAs binding sites) [3–5]. Specifically, the binding of miRNAs to the complementary sequences of the target mRNAs conveys close to the target the RNA-induced silencing complex (RISC) proteins. In summary, the miRNAs exert their specific regulatory functions affecting the stability or translation of targeted mRNA. Importantly, they have been described to partake in numerous cellular processes such as proliferation, differentiation, cellular growth, tissue remodeling, being also implicated in several human pathologies [4]. In mammals, miRNAs are estimated to regulate half of all protein-coding genes being involved in nearly the totality of the cellular functions currently investigated [6]. Several miRNAs have been described as important regulators of liver patho-physiology including liver regeneration, NAFLD, cirrhosis and hepatocellular carcinoma [4,7]. This review describes biogenesis, function, activity and regulation of miRNAs with particular concern for their involvement during NAFLD development and its progression to hepatic fibrosis.

## 2. MiRNA Biogenesis and Function

**Figure 1:**
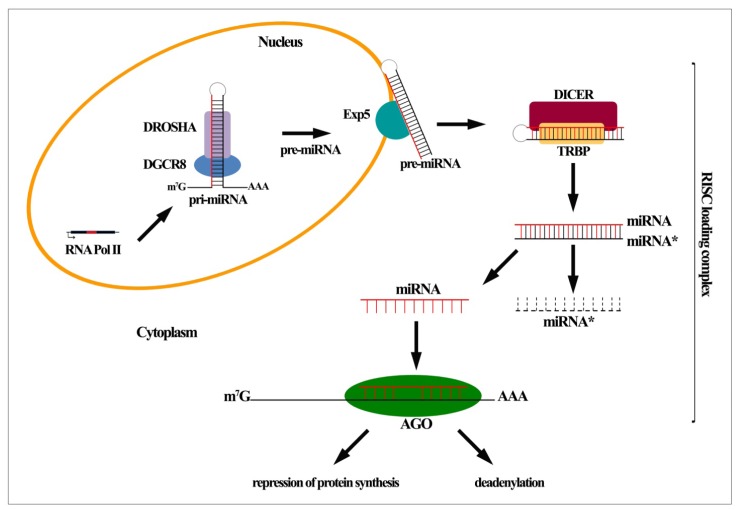
MiRNA (MicroRNA) biogenesis. MiRNAs are typically transcribed in a RNA Polymerase II-dependent manner although alternative pathways exist. The primary precursor (pri-miRNA) is processed into the nucleus by Drosha complex (comprising its cofactor DGCR8) originating the hairpin precursor (pre-miRNA), which is in turn exported in the cytoplasm by the exportin-5 (Exp5). The assembly of the RNA-induced silencing complex (RISC) lead to a second cleavage accomplished by Dicer enzyme (assisted by the dsRNA-binding protein TRBP) giving rise to mature 20–23 nucleotides miRNA:miRNA* duplex. Then the miRNA* strand is released and degraded while the miRNA guide strand is loaded into Argonaute (AGO) complex allowing its association with target RNAs. The incomplete matching of nucleotides among miRNA and target RNAs results in repression of protein synthesis and/or mRNA deadenylation and following degradation.

In contrast to the exogenous long dsRNA precursors generating short interfering RNAs (siRNAs), miRNAs are produced by an endogenous longer primary precursor (pri-miRNA) encoded in the genome and typically transcribed by RNA Polymerase (Pol) II [5]. The resulting dsRNA precursor is polyadenylated and capped as the other RNA Pol II transcripts and presents regions in which the sequences are not perfectly complementary, forming a so-called stem–loop structure [8]. During the canonical miRNA biogenesis pathway the pri-miRNA is processed into pre-miRNA through two ribonuclease (RNase) III-family members that are Drosha and Dicer. Drosha binds and cleaves the dsRNA precursor at the stem-loop structure bearing 60–100 nucleotides RNA precursor (called pre-miRNA). Drosha, in collaboration with its cofactor DGCR8, and other functional proteins (as p68 and p72 helicases) composes the microprocessor complex [5] (Figure 1). Following the first cleavage, exerted by Drosha into the nucleus, the resulting pre-miRNA moves into the cytoplasm transported by the exportin-5 (Exp5)/Ran-GTP complex (Figure 1). Diverse alternative mechanisms, distinguished from the canonical pri-miRNA Drosha-dependent generation, have been described. Some of them are the intronic pri-miRNAs (named “mirtrons”), which are processed by the spliceosome in the nucleus giving rise to pre-miRNAs in Drosha-independent manner. Then, they transferred in the cytoplasm to be further processed and to generate mature miRNAs [9,10]. Whether a given miRNA is generated through the canonical or alternative pathway, once in the cytoplasm, the pre-miRNA undergoes a second cleavage by the RNase III enzyme Dicer, generating a mature 20–23 nucleotides miRNA: miRNA* duplex [11]. The duplex is composed by the guide strand and the passenger (or miRNA*) strand, which each have a different destiny, since the first is loaded into Argonaute (AGO) complex while the latter is released and degraded [6] (Figure 1). In mammals, the Argonaute2 (AGO2) is the core component of the miRNA-induced silencing complex (miRISC), together with its RNase H-like endonuclease activity. The complete RISC loading complex consists also of the Dicer enzyme supported by the dsRNA-binding protein (TRBP in humans) and glycine-tryptophan protein of 182 kDa (GW182) with its downstream repression activity [6,12] (Figure 1). As anticipated, within the cytoplasm the miRISC incorporates the miRNA:miRNA* duplex where AGO2 cleaves and eliminates the non-guide (passenger) strand and then permits the association of the guide miRNA with target RNAs [12]. In mammals, the miRNA-mediated gene silencing is guided by an incomplete matching of nucleotides among miRNA and target RNAs resulting in repression of protein synthesis and/or mRNA deadenylation and consequent degradation [6,12] (Figure 1). Belonging to a diverse mechanism, a perfect match between miRNA and target RNAs, that finally conducts to an endonucleolytic cleavage and degradation, is typical of the regulation of miRISC-dependent gene expression in plants. Although rare, this mechanism can be found also in animals [6].

## 3. MiRNA Regulation, Activity and Decay

Based on the numerous functions exerted in gene expression regulation, in cell physiology and development, and considering their involvement in affecting various diseases, miRNAs are tightly and finely regulated at different stages. The miRNAs can be controlled at transcriptional level, along their multistep processing and post-transcriptionally. Moreover, the miRNA turnover and function can be regulated by differential AGO protein interactions. Furthermore, miRNAs can be controlled by epigenetic modifications and they can even be sequestered from their proper mRNA targets by mean of the so called competing endogenous RNA (ceRNA) [13–15]. Similar to protein-coding genes, miRNA transcription is regulated by conventional regulation structures including CpG islands, TATA box and transcription factor binding sites [6,13]. In addition, the miRNA transcription is correlated to the mRNA expression of the hosting gene because they usually share the leading promoter. However, a considerable amount of intronic miRNAs can be associated to supplementary independent promoters [13]. Thus, the pri-miRNA Pol II-dependent transcription regulation is the most important and the first point of positive and negative control ruled by specific transcription factors, enhancers and silencers. Diversely, the post-transcriptional regulation can act on microprocessor complex processing, pri-miRNA export control, RISC loading complex stability or on the miRNA turnover. By way of example, numerous molecules positively regulate miRNAs, by actingon Drosha microprocessor complexand by interacting with scaffold proteins as p68 and p72 helicases [6,13]. It has been shown that the SMAD proteins regulate the miRNA maturation by Drosha-mediated interaction with conserved motifs on pri-miRNA. Other specific interactions involve the p68 helicase resulting in a SMAD-p68 complex, which induces the miRNA processing and the augmentation of mature miRNAs (as pri-miR-21) [16]. Moreover, after p53 DNA damage-dependent activation, p68 mediates the interaction of p53 with the microprocessor complex, resulting in the modulation of a specific set of miRNAs (as miR-145) involved in the repression of c-myc [17]. Furthermore, accessory proteins promoting Drosha-mediated processing are the splicing factor SF2/ASF, the heterogeneous nuclear ribonucleoprotein A1 (hnRNPA1) and the splicing regulatory protein KSRP. The latter supports the cytoplasmic processing of some pre-miRNAs via Dicer as well [6]. Differently, the nuclear factor NF90-NF45 heterodimer, binding to the pri-miRNA stem, impedes DGCR8 interaction and blocks the pre-miRNA maturation [18]. Another repressor of miRNA processing is the estrogen receptor α(ERα) which prevents both the Drosha complex assembly and the consequent processing of pri-miRNA by the association with p68, p72 helicases and Drosha itself [6,13]. Moreover, the action of adenosine deaminase acting on RNA (ADAR) editing pri-miRNA or pre-miRNA (as for miR-142) represents a different way to impair the miRNA processing altering both mature miRNA balance and miRNA target specificity [19]. Notably, the ADAR-dependent editing is not only restricted to Drosha compartment but it can also intervene within the mature miRNA [20,21]. The microprocessor complex can be repressed by the developmentally regulated RNA binding protein LIN-28, which specifically counteracts let-7 family transcripts cleavage exerted by Drosha. Remarkably, it has been suggested that such regulation is crucial for maintaining the pluripotency state in undifferentiated stem cells being involved in oncogenesis [22]. Furthermore, the pluripotency factor LIN-28 acts as repressor of the pre-miRNA processing affecting Dicer function [23]. The regulator LIN-28, by mean of the terminal poly-(U) polymerase TUT4, permits the polyuridinilation of the let-7 precursor (pre-let-7) decreasing let-7 miRNA in the typical Dicer-guided way. Other targets, as miR-107, -143 and -200c, are repressed by LIN-28/TUT4 [24]. Ablation of Dicer cofactors (as TRBP) is a strategy to modulate Dicer stability and to attenuate pre-miRNA processing [25]. The stability regulation of miRISC by positive and negative modulators depicts an additional level of control of miRNA activity. RNA binding proteins (RBPs) can affect miRISC functions directly, interacting with miRISC molecules, or alternatively associating with miRNA/mRNA duplex. Moreover, the RBPs can represent a scaffold for miRISC interaction with translational repressors or deadenylation effectors, or an obstacle to miRISC/mRNA interaction [6]. In this scenario, the AGO protein levels plays an important role since the augmentation of its protein levels is related to an imbalance of mature miRNAs [26]. Finally, the deregulation of pre-miRNA export and the decrease of mature miRNA amount seem to be a hallmark in human cancers. Indeed, Exp5 inactivating mutations have been found in human tumors with microsatellite instability where the pre-miRNA is blocked in the nucleus and its processing is diminished. Besides, a recovery of the impaired pre-miRNA export has been found to have tumor-suppressor characteristics [27].

## 4. MiRNA Analysis

Since their discovery in 1993, numerous studies have been focused on the analysis of miRNA physiological functions and on their involvement in human diseases leading to the evidence that the miRNA profile alteration associates with several pathologies [28]. Indeed, a wide range of miRNAs are extensively studied because of their valuable diagnostic significance locating the amelioration of sensitivity and specificity of technical approaches for detecting the miRNA expression and modulation in a central position [20]. So far, the most used approach to analyze the expression profile of miRNAs in a certain disease is based on various methods including qRT-PCR: reverse transcription PCR (RT-PCR) followed by quantitative real-time PCR (qPCR), hybridization based methods (microarray analysis) and RNA deep sequencing (RNA-seq) [29]. QRT-PCR methods, based on the reverse transcription of miRNAs into cDNA and next amplification by qPCR, are used to quantify the amplified product sequences. Currently, qRT-PCR is performed both by single miRNA with stem-loop specific primers or by universal primers with poly (A) tail addition. qRT-PCR is the most sensitive method representing a useful validation of the candidate miRNAs identified by more sophisticated and extensive approaches. Nevertheless, the qRT-PCR cannot be used for the identification of novel miRNAs and does not allow an analysis of a big amount of miRNAs [20,29]. The Microarray methodologies are performed in order to compare expression pattern of an amount of known miRNAs at the same time in normal and altered conditions. The RNA samples are hybridized on a microchip, which includes a panel of probes, with high affinity and specificity for different miRNAs. Fluorescence tagged miRNAs are detected by scanning slides and then compared to the standard samples. Despite this profiling permits the contextual analysis of a high number of miRNAs, it is less specific and sensitive than qRT-PCR and RNA sequencing. In addition it cannot furnish an absolute quantity of the targets and it cannot give any information about novel miRNA[20]. The RNA-seq, based on high-throughput next-generation sequencing platforms, is performed by the construction of a cDNA library by reverse transcription of miRNAs to be studied. The library is fixed by an adaptor ligation to a solid phase (Illumina platform, solid phase PCR), or to beads (Roche and ABI platforms, emulsion PCR). The “massively parallel” sequencing gives rise to billions of sequence fragments requiring a bioinformatics analysis which allows to distinguish, with high accuracy, both known and new miRNAs and to furnish an absolute quantification of the RNA samples [20,29]. Diverse companies are trying to reduce the disadvantages of this technology (as the high cost and complex data requiring bioinformatics analysis) developing smaller-scale next generation sequencing platforms. The enormous benefits of the RNA-seq still remain undeniable.

## 5. MiRNAs: Metabolic Functions and Their Implication in NAFLD

It is now extensively accepted the role of miRNAs as potent regulators of a wide range of specific pathways or physiological processes controlling multiple biological functions. Until few years ago the biological functions usually assigned to miRNAs control consisted of developmental timing, apoptosis, proliferation, differentiation and organ development. To date, several recent studies conducted both *in vitro* and *in vivo* models have shown that miRNAs are not only implicated in the regulation of cellular growth and differentiation, but even in the control of energy and hepatic metabolic functions regulating fatty acid (FA) and cholesterol metabolism. Moreover, they are involved in the endoplasmic reticulum stress, oxidative stress, inflammation and apoptosis [30]. Altered expression profiles of miRNAs have been found in several conditions related to metabolic disorders such as obesity, atherosclerosis and diabetes. Recent studies have suggested that differential miRNA expression may have a potential pathological role in deregulation of physiological metabolic pathways, such as insulin resistance and lipid and glucose metabolism, which are all events generally associated with NAFLD pathogenesis and development [30–32]. NAFLD is a well-documented disease currently considered the most common chronic liver disease in industrialized countries, due to hepatic lipid accumulation in the absence of alcohol consumption. This disease presents a characteristic spectrum of liver damage, ranging from simple steatosis to nonalcoholic steatohepatitis (NASH), which can possibly develop towards the more severe liver conditions as fibrosis, cirrhosis and even hepatocellular carcinoma (HCC) [33].

Although NAFLD has been widely recognized as a multi-factorial disease in which a complex interaction of genetics determinants, nutritional factors and lifestyle act in concert to induce hepatocellular injury and progression to liver disease, and despite the enormous amount of ongoing studies, its pathogenesis still remains not fully understood. The emerging role of miRNAs as important players in adipocytes differentiation, hepatic metabolic functions, insulin resistance, appetite regulation and control of the immune response, has led several studies to investigate the relationship between miRNAs altered expression and NAFLD pathogenesis, suggesting some specific miRNAs as potential diagnostic and prognostic markers [4,7]. In particular, the hepatocyte-specific miR-122, which accounts for about 70% of total hepatic miRNAs, is the first, which has been widely associated to metabolic control in liver homeostasis. In the last few years many studies have allowed a better understanding of the critical role played by miR-122 in hepatic lipid metabolism, in cholesterol and FA synthesis modulation. In this regard, the first studies were related to the use of an antisense down regulation technology in mice (antagomirs or antisense oligonucleotides) [34,35]. In mice, the antisense-mediated transient inhibition of miR-122, performed by Esau *et al*. caused an increase in hepatic FA oxidation and a decrease in cholesterol serum levels, in expression of genes involved in cholesterol biosynthesis, including the rate-limiting enzyme 3-hydroxy-3-methylglutaryl coenzyme A (HMGCoA) reductase, and also in hepatic FA and cholesterol synthesis with altered gene expression of fatty acid synthase (FAS) and Acetyl-CoA carboxylases 1 and 2 (ACC1/ACC2) [36]. In addition, miR-122 targeting protected high-fat-fed mice from hepatosteatosis development [36,37]. Similarly, decreased cholesterol serum levels have been observed also in *in vivo* antagonism studies for miR-122 in non-human primates [38]. These initial studies have given a strong contribution for understanding the real involvement of miR-122 in liver lipid homeostasis and its implication in hepatosteatosis or NAFLD. Nevertheless, recent studies on liver-specific long-term deletion of miR-122, displayed different phenotypes with respect to previous transient inhibition studies discussed above. Genetic deletion of miR-122 in germline knockout (KO) mice or liver-specific knockout (LKO) mice showed lower serum cholesterol in accordance with antisense-mediated miR-122 inhibition. However, in young mice the serum cholesterol decrease was associated with an accumulation of hepatic triglycerides, due to an upregulation of genes involved in triglycerides biosynthesis and storage. Among these genes, there are the newly identified miR-122 targets: *Agpat1* (lysophosphatidic acid acyltransferase, alpha) coding an enzyme that converts lysophosphatidic acid into phosphatidic acid, and *Cidec* (cell death-inducing DFFA-like effector c) coding a factor with important roles in apoptosis and lipid droplet formation. This animal model displayed also microsteatosis and liver inflammation events preceding the progression to NASH, fibrosis and tumors HCC-like [39]. Moreover, similar study on mutant mice bearing a prolonged loss-of-function of miR-122a, demonstrated that the animals developed NASH, fibrosis and HCC in adult phase [40]. MiR-122 silencing affected the expression of genes involved in hepatic lipid metabolism that was reflected in increased mRNA of sterol regulatory element binding protein 1-c (SREBP-1c), FAS, and HMGCoA reductase in HepG2 human cell line. Instead, in the same *in vitro* model miR-122 overexpression caused a significant decrease expression of the main lipogenic enzyme genes with a clear impact for better delineating the modulation of these genes in the expression pattern of human NASH [41]. The disagreement of these data with those observed in transient miR122 inhibition animal models studies, can be reconciled considering the possible long term miR-122 suppression needed for establishing a liver pathology [40]. Further, concerning the metabolic key role of the miR-122, it has been reported that miR-122 significantly decreases in NAFLD rat models obtained by different dietetic hypercaloric regimens consisting in a diet added of fats and low in carbohydrates (HFD), a standard diet with high fructose (SD-HF) and a diet high in fats and fructose (HFD-HF) [42].

Another potent post-transcriptional regulator of lipid metabolism appears to be the miR-370. This evidence is suggested by an *in vitro* study in HepG2 cell line in which antisense miR-370 transfection caused an increase in the expression of lipogenic genes such as *SREBP1c*. It is also known that miR-370 directly targets the carnitine palmitoyl transferase 1a (Cpt1a), a mitochondrial enzyme involved in FA oxidation. Interestingly, miR-370 may have a role in hepatic triglycerides accumulation through the modulation of miR-122 expression [43]. So far, there are a growing number of studies which specifically explored the real involvement of miRNAs in the mechanism associated to NAFLD development. In an *in vitro* study by Zheng and colleagues, the steatotic human hepatocyte model L02 cultured with high concentration of free FA, were used for expression profiling of miRNAs. The investigation led to the identification of the miR-10, found significantly altered in steatotic cells, as a potential regulator of steatosis in NAFLD pathogenesis. In particular, the overexpression of miR-10b increased the triglyceride levels and lipid content in steatotic L02 cells by targeting the peroxisome proliferator-activated receptor-α (PPAR-α), a nuclear receptor involved in storage and catabolism of FA and liver inflammation, suggesting miR-10b as potential target for the treatment of NAFLD [44]. Another study investigated the association between the miR-467b modulation and NAFLD. A significant decreased miR-467b expression was observed in liver tissues from high-fat-fed mice (Table 1). Contextually, it has been reported an increase in hepatic mRNA level of lipoprotein lipase (LPL), a predicted target of miR-467b involved in lipid metabolism. The *in vitro* data were confirmed by *in vitro* model of hepatic steatosis stearic acid (SA)-induced. Thus, even the miR-467b can be counted among the miRNAs that play important roles in the development of NAFLD [45]. Hoekstra *et al*. studied the miRNA expression pattern in hypercholesterolemic low-density lipoprotein receptor (LDLR) knockout (−/−) mouse models, which are induced to develop NAFLD by high-fat/Western-type diet (WTD). The miRNA expression profile showed that two specific miRNAs were altered in hepatocytes from mice fed chow or WTD. The WTD diet strongly decreased the expression level of miR-216 (*p* < 0.05) and miR-302a (*p* < 0.01) [46] (Table 1). In particular, the authors reported that the decreased expression of miR-302, induced by high-fat/WTD, was associated with an increase in mRNA expression levels of its target genes, the adenosine triphosphate (ATP)–binding cassette (ABC)-A1 transporter and the long-chain FA elongase (ELOVL6), a microsomal enzymes involved in the formation of long-chain FA [46]. The bibliographical literature, both in mouse and in human studies, indicates that the best known microRNA involved in lipid metabolism and energy homeostasis consists in miR-33a/b, an intronic miRNAs located within the sterol *SREBP-2* and *SREBP-1* genes respectively and co-transcribed with their host genes that are preferentially involved in cholesterol and FA metabolism. SREBP-2 controls cholesterol synthesis and its uptake, while SREBP-1 regulates genes involved in the FA biosynthesis, so that an increased SREBP activity may result in cholesterol and FA accumulation [47]. There are three major genes that have been described as targets of miR-33: The ABC-A1 transporter that mediates cholesterol efflux to lipid-poor apolipoprotein (Apo) A1, the ABC-G1 that is involved in macrophage cholesterol and phospholipids transport, and Niemann Pick (NP)-C1 a protein which regulates the cholesterol transport from lysosomes to other cellular compartments. The use of different approaches for *in vivo* miR-33 inhibition (synthetic inhibitors or genetic deletion) increased the ABC-A1 hepatic expression promoting the reverse cholesterol transport pathway (cholesterol efflux to ApoA1), with consequent raise of circulating HDL levels [48]. These findings were confirmed by non-human primates study where the inhibition of miR-33 expression led to increased plasma HDL levels corroborating the existence of pivotal role of miR-33 in regulating lipid metabolism [49]. More recently, it has been observed that miR-33 fulfills its function of lipid metabolism monitor by the post-transcriptional regulation of genes involved in the β-oxidation of FA, including carnitine *O*-octanoyltransferase (CROT), hydroxyacyl–coenzyme A–dehydrogenase (HADHB), and carnitine palmitoyltransferase 1A (CPT1A). When miR-33a/b were overexpressed, FA oxidation reduction occurred, causing triglycerides accumulation in human hepatic cells and fat body in transgenic flies. In addition to lipid metabolism, overexpression of miR-33 has been shown to reduce insulin signaling pathway, by targeting insulin receptor substrate-2, and consequently reducing the activation of downstream molecules as AKT and ERK [50,51]. In summary, keeping in mind the pivotal role of miR-33 as biosensor of energy homeostasis by controlling lipid metabolism and insulin signaling, it is possible to conjecture that also miR-33 as well as miR-122 and numerous other miRs may have crucial functions in metabolic diseases such as NAFLD and its progression to more severe pathological conditions, opening the possibility for new and promising diagnostic or therapeutics strategies.

Indeed, to better elucidate the miRNAs role in hepatic steatosis and in its eventual progression to steatohepatitis, and in order to highlight an eventual association between alteration of hepatic miRNAs expression pattern and specific stages of NAFLD, Cheung *et al*. analyzed the hepatic miRNAs expression profiles in subjects with metabolic syndrome in presence or absence of NASH diagnosis (defined by enzymes, ultrasound and liver biopsy). From the study, results showed that 46 out of 474 human miRNAs are differentially expressed in subjects with NASH. Among these, miR-34a and miR-146b were significantly increased in liver NASH subjects while in the 63% of the same patients miR-122 was significantly decreased (Table 1) [41]. In a study of biopsy-proven NAFLD and biopsy-proven NASH patients, miR-34a has been found to be involved in the regulation of HMGCoA reductase phosphorylation. Indeed, HMGCoA reductase, which correlates with the dysregulation of synthesis and cholesterol metabolism and with the severity of the NAFLD disease, is increased in expression and dephosphorylated both in NAFLD and in NASH pathological conditions [52]. More in detail, miR-34a, that is overexpressed in NAFLD and NASH, inhibiting sirtuin (SIRT)-1, leads to the AMP kinase (AMPK) dephosphorylation which in turn decreases the HMGCoA reductase phosphorylation. Possibly, miR-34a increased levels in NAFLD can serve to maintain HMGCoA reductase in the active dephosphorylated form thus influencing the hepatic cholesterol accumulation [52]. On the other hand, the circulating levels of miR-34a (together with miR-122 and miR-16) have been examined both in subjects with chronic hepatitis C (CHC) and with NAFLD resulting increased when compared to the controls and even linked with the severity of the diseases. Specifically, in both diseases the miR-34a levels resulted associated with liver enzymes, degree of fibrosis and inflammation activity and they resulted positively related to the NAFLD severity from simple steatosis to NASH [53]. Moreover, the interdependence between miRNAs levels and the individual susceptibility to NAFLD has been explored in NAFLD diet-induced mice from genetically diverse strains. A panel of diverse miRNAs has resulted to be differently expressed in a strain-dependent manner conferring strain-specific susceptibility to liver injury. Notably miR-34a was the one with the highest association with the NAFLD specific pathomorphological features and with its severity [54]. Very recently, in NAFLD patients, an activation of miR-34a/SIRT1/p53 pathway has been described to be correlated with the degree of the disease from steatosis to more severe NASH [55]. MiR-34a, inhibiting SIRT-1, leads to the increment of p53 acetylation and transcription conducting to expression of pro-apoptotic genes and then apoptosis suggesting miR-34a as a possible regulator of hepatocytes apoptosis during NAFLD. Further, it has been shown that in rat livers and in cultured primary hepatocytes the ursodeoxycholic acid (UDCA) specifically inhibited the miR-34a/SIRT1/p53 pathway. In addition, UDCA significantly decreased the *in vitro* miR-34a overexpression in primary rat hepatocytes transfected with miR-34a precursor simulating the more advanced NAFLD condition [55]. Given the strong correlation of miR-34a amount with the NAFLD severity and its increasingly evident influence on its pathogenesis, the miR-34a/SIRT1/p53 pathway arises as a potential target to ameliorate NAFLD severe condition.

Actually, several studies carried out in animal models have repeatedly confirmed the existence of a relationship between the expression profile of diverse miRNAs and the specific disease stage of NAFLD. Jin *et al*. established a rat model of NAFLD fat-rich-diet-induced in which hepatic miRNAs expression profiles in different stages of the pathology were evaluated by microarray analysis and stem-loop RT-PCR. The authors found 58 miRNAs up-regulated and 51 miRNAs down-regulated in the steatosis and steatohepatitis group [56]. Another study demonstrated that the changes in miRNAs expression are strongly associated with the severity of NASH in mice. Besides, NASH animals’ livers from C57BL/6J and DBA/2J mice fed with methyl-deficient diet, displayed a specific differential miRNAs expression pattern with respect to the control group. MiR-34a, miR-155, miR-200b and miR-221 were found overexpressed while miR-29c, miR-122, miR-192 and miR-203 were downregulated in both C57BL/6J and DBA/2J mice, with a greater effect in DBA/2J mice [57] (Table 1). More recently, it has been also demonstrated a downregulation of miR-27, miR-122, miR-451 and an upregulation of miR-200a, miR-200b and miR-429 in NAFLD rat models fed with HFD, SD-HF or HFD-HF. Furthermore, this alteration in miRNAs expression resulted in correlation with diverse histological traits and metabolic altered parameters distinguishing the NAFLD condition [42] (Table 1). The ability to attribute a specific spectrum of expression to each stage of NAFLD would represent an important breakthrough for the identification of new potential diagnostic or therapeutic tools.

## 6. MiRNAs and Their Implication in Hepatic Fibrosis

NAFLD condition affects one-third of the adult population in Western countries and it is also increasing along the childhood in the same geographic area. The clinical importance of this pathology results clear since, in a fashion still poorly understood, simple steatosis can progress to steatohepatitis, characterized by hepatocellular inflammation, and possibly evolve to end-stage liver disease and complications as hepatic fibrosis [58,59]. Notably, the progression of liver fibrosis comprises cirrhosis development, characterized by distortion of the normal tissue architecture, formation of regenerative nodules surrounded by septae, portal hypertension, hepatocellular carcinoma, and eventually liver failure [60]. In the past years, several studies revealed human hepatic stellate cells (HSCs) as the main pro-fibrogenic cell in the liver. Chronic hepatic injuries trigger HSCs to switch from a quiescent state to an active myofibroblastoid phenotype, marked mainly by the induction of α-smooth muscle actin (α-SMA), increased cell proliferation rate, extracellular matrix (ECM) degradation, chemotaxis and inflammatory signaling. Activated HSCs, secreting a large quantity of ECM proteins and fibrillar collagens at the damaged sites, play a major role in tissue remodelling and fibrosis [61]. Despite its clinical importance, the mechanism of hepatic fibrosis is still not completely understood and few effective therapies are available up to now. Noteworthy, diverse studies have shown an involvement of the miRNAs in the process of fibrogenesis and modulation of HSCs activation, which will be progressively examined below. Given the huge number of pathways affected by miRNAs, the investigation of the role of miRNAs in liver fibrosis could be helpful to provide new therapeutic and also diagnostic tools for the common condition of hepatic fibrosis. In order to analyze the possible association between miRNAs expression pattern and the progression of liver fibrosis, Murakami *et al*. examined the expression profile of miRNAs in a mouse chronic liver inflammation model and in human clinical biopsies by miRNA microrray analysis [62]. The mice study revealed that 11 miRNAs were related to the progression of liver fibrosis (mmu-let-7e, miR-125-5p, -199a-5p, -199b, -199b*, -200a, -200b, -31, -34a, -497, and -802). Interestingly, 4 miRNAs (miR-199a, -199a*, -200a, and -200b), sharing the sequence between human and mouse, showed a similar pattern of expression both in the mouse and in the human study, correlating positively and significantly with the grade of fibrosis [62] (Table 1). In a rat model study on dimethylnitrosamine (DMN)-induced fibrosis, a miRNA array analysis revealed the progressive upregulation of 16 miRNAs (the 10 most upregulated miRNAs were miR-34b, -34c, -34a, -221, -146b, -214, -199a-5p, -199a-3p, -223, -324-5p) and downregulation of 7 miRNAs (the 3 most downregulated miRNAs were miR-378, -193, -878) in association with the progression of hepatic fibrosis, as compared to the control group [63] (Table 1). In particular, miR-34 family, the most upregulated one along hepatic fibrosis development, may be involved in lipid/FA metabolism, by targeting acyl-CoA synthetase long-chain family member 1 (ASCL1) [63]. In a recent study, Roderburg *et al*., found that 31 miRs were differentially regulated in a model of CCl_4_-induced fibrosis. Among these, the miR-29 family members showed a significantly down-regulation when compared with non-fibrotic livers both in different animal models of hepatic fibrosis and in human samples of advanced fibrosis, being also lower at serum level in patients with advanced liver cirrhosis if compared with patients at the early stage of fibrosis [64]. Recently, a study investigated the role of miR-214-5p in hepatic fibrogenesis using both *in vivo* and *in vitro* experimental models. The authors found that miR-214-5p was upregulated in a fibrosis progression-dependent manner in the livers of patients with chronic hepatitis C virus (HCV) infection and in mice with diet-induced steatohepatitis. Moreover, miR-214-5p expression increased during the culture-dependent activation of mouse primary stellate cells. Additionally, in hepatic stellate cell line LX-2 the overexpression of miR-214-5p increased the expression of fibrosis-related genes, such as matrix metalloproteinase (as MMP-2 and MMP-9), α-SMA and transforming growth factor transforming growth factor (TGF)-β1 [65] (Table 1). In recent years, miRNAs have been recognized to be involved in regulating HSCs activation, proliferation or apoptosis. In activated rat HSCs, it has been shown a change in miRNAs expression identifying 12 up-regulated (miR-874, -29c*, -501, -349, -325-5p, -328, -138, -143, -207, -872, -140, 193) and 9 down-regulated miRNAs (miR-341, -20b-3p, -15b, -16, -375, -122, -146a, -92b, -126) [66] (Table 1). Similar findings have been reported using miRNAs microarray analysis in partially and fully activated rat HSCs compared to quiescent HSCs [67]. A study conducted by Ji *et al*. reported that down-regulation of miR-27a and 27b promotes culture-activated rat HSCs to switch to a quiescent phenotype, characterized by cytoplasmic lipid droplets and decreased cell proliferation rate, losing morphological features of activated cells. Further, it has been experimentally confirmed that retinoid X receptor α (RXRα), involved in many signaling pathways related to cell proliferation, differentiation and adipogenesis, is the target of miR-27a and 27b [68]. MiR-150, miR-194 and miR-29b also appeared to be involved in HSCs trans-differentiation in rat and human experimental models respectively [69,70]. It is widely recognized that spontaneous or induced HSCs apoptosis is associated with regression of liver fibrosis in animal model and patients with chronic liver disease [71,72], thus there is an increasing interest in depicting the mechanisms related to this process. On this purpose, in the last few years some studies identified a role for miR-15, miR-16 and miR-181b in the regulation of cell cycle progression and in the apoptosis induction in HSCs [73]. A comparative bioinformatics analysis revealed that in quiescent and activated HSCs 13 signal transduction pathways were upregulated and 22 were downregulated by miRs. Furthermore, the mitochondrial pathway of apoptosis seems to play an important role in the progression of liver fibrogenesis, via HSCs activation, with BCL-2 and caspase-9 regulated by miR15b/16 and miR-138 respectively [74].

According to the literature, the cytokine TGF-β1 plays a key role in triggering the trans-differentiation of HSCs into fully activated and pro-fibrogenic phenotype, thus causing hepatic fibrosis. In a recent paper He *et al*. investigated the involvement of miR-146a in TGF-β1-treated HSCs activation, reporting that overexpression of miR-146a was able to inhibit TGF-β1-induced HSCs proliferation and to increase cell apoptosis [75]. It is well accepted that the activation of HSCs is in part caused by the inflammatory process, during which different cytokines are secreted by various liver cells like hepatocytes, Kupffer cells and endothelial cells [76,77]. Maubach and co-workers have shown that interleukin-1 receptor-associated kinase (IRAK)-1 and tumor necrosis factor (TNF) receptor-associated factor (TRAF)-6, two crucial upstream factors leading to nuclear factor (NF)-κB pathway activation, were downregulated by overexpression of miR-146a in an *in vitro* model of HSCs. Moreover, miR-146a overexpression upregulated the tissue inhibitor of metalloproteinases (TIMP)-3 mRNA, a crucial regulator of the inflammatory cytokine TNFα, suggesting the idea of an association between miR-146a, TNFα activity and inflammation process. The authors also showed the involvement of miR-26a, 29a and 214 in the regulation of collagen type I mRNA [78]. In the near future, the numerous pathways affected by miR-146a overexpression and the mechanism behind miR-146a regulation in liver fibrosis need to be further investigated.

Despite its well-known antivirus effect, a number of publications reported that interferons (IFNs) also exhibit an antifibrotic action in human chronic hepatitis [79,80]. Using the HSCs *in vitro* model LX-2, Ogawa *et al*. demonstrated that IFN can induce the upregulation of miR-29b, which can negatively regulate the expression of type I collagen by the direct interaction of mir-29b to Col1A1 3′UTR [70]. Recently, it has been found that IFN-β is able to induce miR-195, whose overexpression increased p21 mRNA and protein levels, lowered cyclin E1 mRNA and protein levels and inhibited cell proliferation in LX-2 cells [81]. These results reveal a new mechanistic aspect of the antifibrotic effect of IFNs in the liver suggesting new therapeutic strategy for liver fibrosis.

## 7. Concluding Remarks

Nowadays, the significance of miRNAs regulation in different physiological and pathological conditions is becoming increasingly visible and undeniable. A quantity of studies have reported the crucial roles that miRNAs exert along the onset and development of various pathologies including acute and chronic liver diseases as NAFLD, NASH, fibrosis, cirrhosis and also HCC. In particular, aberrant miRNAs expression has been demonstrated to affect various aspects of metabolic pathways, with particular regard to lipid and glucose metabolism, oxidative stress, inflammation and apoptosis. Moreover, another significant emerging aspect is that the serum level of certain liver-specific miRNAs might be used as clinically potential biomarkers since augmentation in circulating concentration of specific miRNAs have been related to different stages of NAFLD, NASH and to the progression of fibrotic liver damage [7,20,53,64]. Furthermore, miRNAs have emerged as potential targets for the treatment of liver pathological conditions [4,82], which is a fascinating new area to be explored, not forgetting some limitations due to the complex regulatory interplay caused by the multispecificity of a single miRNA. From a side there is the functional analysis of the miRNAs mechanisms regulating NAFLD and its progressive hepatic complications. Indeed, the study of the relationship between the miRNAs expression pattern and the clinical stages of the disease remains fundamental in order to depict additional non-invasive clinical markers. From the other side the identification of potential miRNAs targets and their possible use as new miRNA-based therapeutic products will be open issues for the future.

## Figures and Tables

**Table 1:** Descriptive summary of altered miRNAs in several hepatic settings.

Condition	HUMAN		MOUSE/RAT		References
			
	Upregulated miRs	Downregulated miRs	Upregulated miRs	Downregulated miRs	
NAFLD/NASH	10b, 16, 29c, 33, 34a,122 (circ), 146b	99b, 122 (liver), 132, 150, 511a	MOUSE: 34a, 155, 200b, 214-5p, 221 RAT: 16, 10b, 29c, 33, 34a, 122, 200a/b, 429	MOUSE: 29c, 122, 192, 203, 467b, 216, 302a RAT: 27, 29b, 122, 203, 451	[4,20,41,42,45,46, 52–55,65]

FIBROSIS	34c, 125-5p, 199a/b, 200a/b, 221, 223	29a/b/c, 30b/c, 96, 132,193, 341, 183, 877	MOUSE: mmu-let-7e, 31, 34a, 125-5p,199a-5p, 199b, 200a/b, 497, 802 RAT: 34a/b/c, 214, 221, 146b, 199a-5p, 199a-3p, 223, 324-5p	MOUSE: 29a/b/c RAT: 193, 378, 878	[4,62–64]

Activated HSCs	214-5p	29b, 150, 194,	MOUSE: 214-5p RAT: 138, 140, 143, 193, 207, 501, 325-5p, 328, 349, 872, 874	RAT: 15b, 16, 20b-3p, 27a/b, 29b 92b, 126, 122, 146a, 150, 194, 341, 375	[65,66,69,70,75]

NAFLD: nonalcoholic fatty liver disease; NASH: nonalcoholic steatohepatitis; HSCs: hepatic stellate cells; miRs: microRNA.
